# Persisting Gastrointestinal Symptoms in Children with SARS-CoV-2: Temporal Evolution over 18 Months and Possible Role of Lactoferrin

**DOI:** 10.3390/children11010105

**Published:** 2024-01-15

**Authors:** Francesco Mariani, Saveena Rainaldi, Giulia Dall’Ara, Cristina De Rose, Rosa Morello, Danilo Buonsenso

**Affiliations:** 1 Department of Woman and Child Health and Public Health, Fondazione Policlinico Universitario A. Gemelli IRCCS, 00168 Rome, Italy; francescomariani@omceoromapec.it (F.M.); cristina.derose@guest.policlinicogemelli.it (C.D.R.); rosa.morello1@guest.policlinicogemelli.it (R.M.); 2 Medicine and Surgery International Faculty, Università Cattolica del Sacro Cuore, 00168 Rome, Italy; saveena.rainaldi01@icatt.it (S.R.); giulia.dallara01@icatt.it (G.D.); 3 Centro di Salute Globale, Università Cattolica del Sacro Cuore, 00168 Rome, Italy

**Keywords:** SARS-CoV-2 infection, long COVID, post COVID condition (PCC), children, gastrointestinal symptoms, lactoferrin

## Abstract

Background and aim: Persisting gastrointestinal symptoms are reported to be relatively common in children with long COVID; however, their detailed characterization and long-term outcomes have not yet been described. Methods: We performed a retrospective study aiming to investigate the temporal evolution of gastrointestinal symptoms in children with SARS-CoV-2, from acute infection to 18-months follow-up. To further investigate possible therapeutic strategies, we evaluated the role of lactoferrin in improving gastrointestinal symptoms in these children, compared with those not treated. Results: A total of 1224 patients (47.7% females) were included. Of these participants, 246 (19.8%) were vaccinated and 143 (11.5%) presented with comorbidities. A total of 175 patients (14.1%) presented gastrointestinal symptoms during acute infection, 54 (4.4%) at three months, 23 (1.9%) at six months, 6 (3.3%) at twelve months, and 2 (2.3%) at eighteen months follow-up. At six months follow-up, children who were treated with 3 months oral lactoferrin had less persisting symptoms compared to those who did not receive lactoferrin, although this difference was not statistically significant (three patients (25%) in the lactoferrin group vs. fourteen patients (33.3%) not treated, *p* = 0.73), probably due to the low number of patients with persisting GI symptoms. Conclusions: GI symptoms are relatively common during acute SARS-CoV-2 infection in children, and a non-negligible proportion of these children reported persisting symptoms for up to 12–18 months after the acute infection. In addition, we found a trend even if statistically nonsignificant toward faster improvement of persisting gastrointestinal symptoms in children with long COVID treated with lactoferrin. Despite the limitations relating to the present study’s design, given the significant burden of gastrointestinal symptoms in children with long COVID, our findings provide the basis to perform a prospective, placebo-controlled study.

## 1. Introduction

Long COVID, post-COVID condition (PCC) is a long-term consequence of SARS-CoV-2 infection that can affect both adults and children [1]. Estimates suggest that about 40% of patients after an acute infection develop at least one of the symptoms of long COVID syndrome [2]. In relation to the pediatric population, long COVID symptoms seem to affect one-quarter of children, even at one year after infection [3]. Long COVID is a heterogeneous entity with a broad spectrum of clinical manifestations. In most children, more than one organ system is involved, especially the respiratory, cardiovascular, neurocognitive, musculoskeletal, and gastrointestinal systems [4].

In particular, the gastrointestinal (GI) system appears to be a specific target for SARS-CoV-2. In children, GI symptoms have been frequently reported across the whole spectrum of the infection, from acute infection to post-COVID complications including the multisystem inflammatory syndrome and long COVID [5]. The pathophysiology of acute and chronic GI symptoms in children infected with SARS-CoV-2 is not yet precisely known. Putative mechanisms of pathogenesis are the persistence of SARS-CoV-2 in the gut, stimulating local inflammation, persistent subcellular damage, immunity-triggered inflammation, and alteration of the microbiota [6]. Also, GI inflammation can lead to dysfunctional signaling in the brainstem and/or vagus nerve [7,8,9], which in turn contributes to dysautonomia and postural orthostatic tachycardia syndrome (POTS) [10,11].

However, despite several advances that have been performed in the understanding of long COVID in adults, pediatric research has still fallen behind, and the temporal evolution of GI symptoms over time in children remains unknown. Moreover, the optimal management of chronic GI symptoms is still unknown; however, given the mentioned candidate pathogenetic mechanisms, some strategies have been hypothesized as therapeutic possibilities, such as probiotics [12] and lactoferrin [13].

Given these considerations, we have retrospectively described a cohort of children with long COVID characterized by GI symptoms seen in a pediatric post-COVID outpatient clinic, and evaluated the role of lactoferrin, which was initially used in the clinic for the treatment of these symptoms, given its known immunomodulatory effects on the gut mucosa.

## 2. Materials and Methods

### 2.1. Study Population and Setting

This is a retrospective sub-analysis of a cohort of pediatric patients (<18 years of age) evaluated in the post-COVID outpatient clinic in our third-level hospital in Rome, Italy. At the beginning of the pandemic, we developed a protocol to identify children with persistent symptoms after acute SARS-CoV-2 infection and hypothesized a personalized diagnostic approach [14]. Children with SARS-CoV-2 infection seen in our hospital (either inpatient or emergency department, or from primary care pediatricians in the catchment area) were offered a follow-up about 12 weeks (three months) after the initial diagnosis of the infection, independently from the severity of acute disease. When assessed at 12 weeks follow-up, in case of the persistence of GI symptoms at three months after the initial infection, patients underwent further examinations to exclude other diagnoses (such as anemia, celiac disease, autoimmunity, thyroid disease, and parasitic infections). In the absence of other possible causes, children with persistent GI symptoms for at least three months after the initial infection, which impaired daily life, were diagnosed with long COVID according to the definition provided by the World Health Organization [15]. On the contrary, children who were fully recovered after 12 weeks and reported no complaints were classified as “fully recovered” from SARS-CoV-2 infection. All children were assessed at 3–6–12–18 months following SARS-CoV-2 acute infection, as part of a study that will include also 24- and 36-months follow-up. The same group of pediatricians in charge of the post-COVID clinic evaluated patients during follow-ups. This particular study includes children evaluated from February 2020 to October 2022. Further details about the organization and procedures of our pediatric post-COVID unit are available elsewhere [16].

Initially, in our post-COVID unit and in patients with persistent and disabling GI symptoms, oral lactoferrin was prescribed for 90 days (at a dosage of either 600 mg/die (if younger than 8 years old) or 800 mg/die (if aged 9 to 17 years of age) for 90 days). Lactoferrin’s complete composition is: lactoferrin; corn maltodextrin; anticaking agents (magnesium salts of fatty acids, silicon dioxide); capsule composed of hydroxypropyl methylcellulose. The cohort of children with GI symptoms who received lactoferrin was compared with those children who did not receive this drug (e.g., in the case of parental or patient refusal).

### 2.2. Inclusion and Exclusion Criteria

The following inclusion criteria were met: children aged 0–18 years; children referred to our outpatient clinic during the study period; a laboratory (RT-PCR, COVID-19 antigen tests or SARS-CoV-2 antibody testing) confirmed SARS-CoV-2 infection; first assessment 90 days after the diagnosis of the COVID-19 infection; the parent’s/caregiver’s/guardian’s consent to participate

The exclusion criteria were patient aged >18 years; children referred to our outpatient outside the study period; non-laboratory confirmed/suspected SARS-CoV-2 infection; the absence of parent’s/caregiver’s/guardian’s consent to participate.

### 2.3. Primary and Secondary Outcomes

The primary outcome was to describe the temporal evolution of GI symptoms from acute SARS-CoV-2 infection to the point of 18-month follow-up. The secondary outcome was to describe the possible effects of lactoferrin on improving GI symptoms at 6 months follow-up in those children experiencing persisting GI symptoms at three months follow-up.

### 2.4. Statistical Analysis

Categorical variables were reported as count and percentage. The statistical association between categorical variables was obtained by Chi-squared tests or Fisher’s exact tests *p* value < 0.05 was considered statistically significant if not differently specified.

Statistical analysis was performed using IBM SPSS Statistics 28.0 software (IBM Corporation, Armonk, NY, USA).

## 3. Results

### Study Population

**Figure 1:**
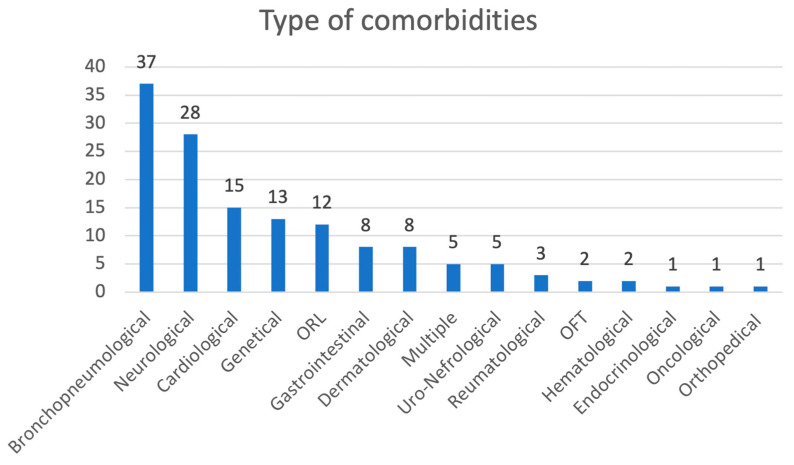
Comorbidities of the study population.

The medical records of 1224 patients (47.7% females) were collected. A total of 246 patients (19.8%) were vaccinated and 143 (11.5%) presented with comorbidities. During the acute infection, 175 patients (14.1%) presented with GI symptoms; in Table 1 details of the study population are reported, while Figure 1 represents the comorbidities of the study population. The percentage of GI symptoms’ persistence in the different follow-up was very low (range 0–4.34%) with a progressive reduction over time, although a very small number of patients (only three) had a 2 years follow-up. Children who needed lactoferrin at follow-up had a higher probability of already experiencing GI symptoms during the acute phase of the infection (*p* = 0.008).

The GI symptom most frequently reported was abdominal pain at the three month follow-up (34 patients, 2.73%), diarrhea at the six month follow-up (10 patients, 0.82%) and at the 12 month follow-up (4 patients, 2.22%), and diarrhea and vomiting at the 18 month follow-up (1 patient, 1.16%) (Table 2). Four patients who were taking lactoferrin since the previous timepoint were still experiencing persisting GI symptoms at the next follow-up.

**Figure 2:**
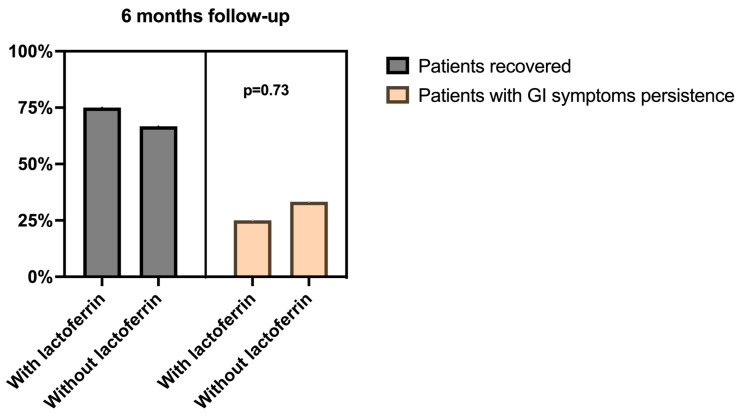
Association between gastrointestinal symptoms (GI) at six months follow-up and lactoferrin administration since one month at that follow-up evaluation.

Considering only the patients who presented GI symptoms at the three month follow-up (*n* = 54), although the cohort patients in the lactoferrin group had a lower percentage of GI symptoms’ persistence at the six month follow-up (3/12 patients, 25%) as compared to those patients not treated (14/42 patients, 33.3%), this association was not statistically significant (*p* = 0.73) (Figure 2).

## 4. Discussion

To our knowledge, this is the first study that has investigated the long-term evolution of GI symptoms in children with SARS-CoV-2 and the role of lactoferrin in improving them. Overall, we found a trend but not a statistically significant one toward faster improvement in persisting GI symptoms in children with long COVID treated with lactoferrin due to persisting GI symptoms.

Lactoferrin has well-known antimicrobial, anti-inflammatory, antioxidant, and immunomodulating properties. Due to its several biological effects, lactoferrin was initially proposed as a possible therapeutic or preventative medication for COVID-19 and, later, also for long COVID, for gastrointestinal diseases in children [13].

Lactoferrin has immunomodulatory effects and stimulates cells involved in both innate and acquired immunity, but also increases immunity against viral and bacterial diseases in humans and animals [17,18]. In addition, lactoferrin has effects on plasminogen which is essential for the degradation of fibrin clots, activation of growth factors, removal of protein aggregates, and cell migration [19], all events thought to be implicated in long COVID. These latter effects are particularly interesting in light of the growing evidence of the pro-coagulatory effects of SARS-CoV-2 both during acute (COVID-19) or chronic (long COVID) infection [20,21,22]. More specifically, regarding the potential chronic gastrointestinal persistent infection which has also been demonstrated in children [23], an in vitro study has examined lactoferrin’s protective effect against SARS-CoV-2, in SARS-CoV-2-infected Caco2 intestinal cells, treated or not with lactoferrin. With the results of qRTPCR, the authors found that lactoferrin may partially inhibit SARSCoV-2 infection [24], which theoretically may be assessed in patients with chronic GI symptoms during long COVID. In studies carried out on adults with SARS-CoV-2 infection, lactoferrin showed an excellent safety and tolerability profile, making its use at least feasible in the context of long COVID [25], where treatment options are currently not available. This is also true for children, where lactoferrin has been extensively studied in several pediatric and neonatal diseases, affecting either the gastrointestinal or respiratory tract, and related or not to viral infections, and always providing optimal safety [26].

Persistent GI symptoms are relatively common in both adults and children [5,14,27], and in this regard lactoferrin is interesting due to its specific roles within the gut [28]. Lactoferrin is a selective modulator of the gut microbiome in animals and humans [29,30,31], through the elimination of pathogenic bacteria and increasing the beneficial ones, such as bifidobacterial and lactobacilli, eventually preventing gut dysbiosis. In a recent randomized, double blind, placebo-controlled, multicentric trial, the impact of oral supplementation of lactoferrin on the gut microbiome was evaluated in pediatric oncohematologic patients during chemotherapy. After two weeks of treatment, compared with placebo, lactoferrin was safely administered with no adverse effects and promoted gut microbiota homeostasis by favoring the maintenance of diversity and preventing the overgrowth of pathobionts (e.g., Enterococcus) [32].

It is suggested that, during COVID-19, the gut microbiota is involved in the contribution of disease severity via modulating host immune responses. In addition, gut dysbiosis after disease resolution may contribute to lasting symptoms, emphasizing a need to understand how the gut microbiota is involved during COVID-19 [33,34,35,36]. Also, lactoferrin is able to promote the proliferation of gastrointestinal cells [37]. In humans, lactoferrin supplementation reduced a drug-induced increase in gut permeability and hence may provide a nutritional tool in the treatment of permeability-associated illnesses [38]. These data are extremely important if we consider that impaired gut permeability has been demonstrated in children with multisystem inflammatory syndrome (MIS-C) [39], which has led US researchers to their current investigation of the role of a medication that improves gut permeability to ameliorate the impact of long COVID in adults and children [40].

Given the evidence from this body of work in the literature, we wanted to investigate the role of lactoferrin to improve persistent GI symptoms in children affected by long COVID. So far, there is one registered trial evaluating the role of lactoferrin in the treatment of long COVID, but it is only enrolling patients aged 18–60 years of age, having the primary outcome of faster fatigue improvement in patients treated with lactoferrin (trial ID NL9742) [28]. Therefore, our study is, in our knowledge, the first to describe in detail the pattern of persisting GI symptoms in children with long COVID and to attempt to preliminarily explore if lactoferrin may play a role in children with long COVID characterized by a predominant GI phenotype, as we have previously described that long COVID can manifest with different main clusters of symptoms [14].

We found that persisting GI symptoms are relatively common in children during the whole spectrum of the infection. During acute disease, 14.1% of our large cohort reported at least one GI symptom, which translates to hundreds of thousands of children having GI symptoms on a global scale during the stage of acute infection, despite SARS-CoV-2 being traditionally considered a respiratory virus. Such a finding reinforces previous mentions of a specific GI trophism for this virus, which in turn can facilitate post-acute consequences through local persistent inflammation or alteration in the gut microbiota. As a consequence, it is not surprising that up to 4% of infected children still reported GI symptoms at the 3 month follow-up; again, this represents a significant impact if we translate these percentages of the large number of children infected globally. Despite most patients improving over time, 2% of the children followed-up at 18 months still reported GI symptoms; thus, highlighting the need to develop treatment strategies. Overall, chronic stool abnormalities and abdominal pain were the most described symptoms, which can negatively impact on quality of life for children, independently of their link with SARS-CoV-2.

Of note, the role of lactoferrin may not be limited to the GI and immune effects, since lactoferrin has been associated with lipid and energy metabolism [41,42], and with bile acids [42]. Although the etiology of long COVID is not yet entirely understood, dysregulated metabolism may play a role. Lipid metabolism is highly influenced by COVID-19 [43,44], and bile acids are also dysregulated [45], with existing acute-stage treatments only partially able to modulate these lipid metabolism effects [46]. Therefore, these concepts further reinforce the theorical need of trialing lactoferrin in this condition.

In this context, attempting a safe medication like lactoferrin was deemed favorable due to the lack of other options and in light of the evidence of the safety and tolerability of this medication. Overall, we found that when lactoferrin was started at 3 months, less children had persisting GI symptoms at 6 months when compared with those who did not take lactoferrin, although the differences were not statistically significant, probably also because of the low number of children included in the two groups at the 3 month follow-up. Given the low number of children with persisting GI symptoms at the other timepoints, we could not make any further comparisons. In any case, given the potential rationale for lactoferrin, and the significant impact of chronic GI symptoms in children, we believe that our study opens to the possibility to design prospective, randomized trials in children with long COVID characterized by GI symptoms.

This study has limitations to address. It is a retrospective study and not a placebo-controlled randomized trial. Another consideration might be that lactoferrin was used as a therapeutic strategy already in children infected with the pre-omicron variant, which was an independent risk factor for persistent symptoms [16]. Also, we could not objectively measure adherence to treatment in all patients. As the medication was prescribed for a relatively long period, it is possible that some children may have had low compliance which may have negatively affect the efficacy of the medication. In addition, given the relatively small cohort of children with persisting GI symptoms who received lactoferrin, we could not develop more complex statistical analyses, including Cox proportional hazard methods. Finally, the choice of lactoferrin dosage was based on dosages used in other pediatric conditions, and we cannot exclude that different dosages tailored considering the age and the seriousness of long COVID might need to be used.

## 5. Conclusions

In conclusion, we found that GI symptoms are relatively common during acute SARS-CoV-2 infection in children, and a non-negligible proportion of these children report persisting symptoms for up to 12–18 months after the acute infection. In addition, in our preliminary retrospective observation, we found a clinical trend, even if statistically nonsignificant, toward faster improvement in persisting GI symptoms in children with long COVID treated with lactoferrin due to persisting GI symptoms. Further studies, in larger populations and with a prospective, placebo-controlled design, are needed to determine the therapeutic role of lactoferrin in children, either in the treatment of long COVID, or as an early treatment in children at risk of developing long COVID as a preventative therapeutic approach.

## Figures and Tables

**Table 1:** Study population and comparison between patients receiving lactoferrin for at least one month at 6 months evaluation and patients who did not receive lactoferrin for at least one month at 6 months evaluation. *p* values from chi square or Fisher’s exact test.

	Study Population (*n* = 1244)	Without Lactoferrin for at Least Three Months at 6 Months FUP (*n* = 1186)	With Lactoferrin for at Least Three Month at 6 Months FUP (*n* = 58)	*p*
**Female,** *n (%)*	594 (47.7%)	560 (47.2%)	34 (58.6%)	0.09
**Vaccinated,** *n (%)*	246 (19.8%)	227 (19.1%)	19 (32.8%)	0.01
**Comorbidities,** *n (%)*	143 (11.5%)	133 (11.2%)	10 (17.2%)	0.16
**Severity acute COVID**, *n (%)* Asymptomatic Mild Moderate	108 (8.7%) 1113 (89.5%) 23 (1.8%)	107 (9%) 1058 (89.2%) 21 (1.8%)	1 (1.7%) 55 (94.8%) 2 (3.4%)	0.11
**COVID variant,** *n (%)* *Alfa* *Delta* *Omicron* *Wild*	*72 (5.8)* *246 (19.8)* *889 (71.5)* *37 (3.0)*	70 (5.9%) 231 (19.5%) 849 (71.6%) 36 (3.0%)	2 (3.4%) 15 (25.9%) 40 (69.0%) 1 (1.7%)	0.55
**Hospitalized,** *n (%)*	26 (2.1%)	25 (2.1%)	1 (1.7%)	1
**Intensive care unit hospitalization,** *n (%)*	1 (0.1%)	1 (0.1%)	0 (%)	1
**Gastrointestinal symptoms during acute infection,** *n (%)*	175 (14.1%)	160 (13.5%)	15 (25.9%)	0.008

**FUP:** Follow-up.

**Table 2:** Pattern of persisting gastrointestinal symptoms, with or without the use of lactoferrin.

	3 Month Follow-Up (*n* = 1244)	6 Month Follow-Up (*n* = 1223)	12 Month Follow-Up (*n* = 180)	18 Month Follow-Up (*n* = 86)	24 Month Follow-Up (*n* = 3)
**GI symptoms,** *n (%)*	54 (4.34%)	23 (1.88%)	6 (3.33%)	2 (2.32%)	0 (0%)
**Patients with GI symptoms taking Lactoferrin since three months,** *n (%)*	0 (0%)	3 (0.3%)	1 (0.5%)	0	0
**Nausea,** *n (%)*	10 (0.80%)	4 (0.25%)	2 (1.1%)	0 (0%)	0 (0%)
**Diarrhea,** *n (%)*	13 (1.04%)	10 (0.82%)	4 (2.22%)	1 (1.16)	0 (0%)
**Abdominal pain,** *n (%)*	34 (2.73%)	9 (0.73%)	0 (0%)	0 (0%)	0 (0%)
**Stool abnormalities,** *n (%)*	5 (0.40%)	1 (0.08%)	0 (0%)	0 (0%)	0 (0%)
**Poor feeding,** *n (%)*	4 (0.32%)	1 (0.08%)	0 (0%)	0 (0%)	0 (0%)
**Gastroesophageal reflux,** *n (%)*	2 (0.16%)	2 (0.16%)	0 (0%)	0 (0%)	0 (0%)
**Vomit,** *n (%)*	1 (0.08%)	1 (0.08%)	0 (0%)	1 (1.16%)	0 (0%)
**Other symptoms,** *n (%)*	1 (0.08%)	0 (0%)	0 (0%)	0 (0%)	0 (0%)

## Data Availability

The data presented in this study are available on request from the corresponding author. The data are not publicly available due to specific ethical and privacy considerations.
